# Circumcision in the Adult

**Published:** 1888-04

**Authors:** 


### CIRCUMCISION IN THE ADULT.

Mr. CHARLES A. BALLANCE says, in a communication in *St. Thomas' Hospital Reports*, Vol. XVI, that the pain and discomfort that follow circumcision in the adult are often very considerable. It has occurred to him to use in this operation the same dressing that it is customary to use after experimental lesions in animals, when every effort is required in order to obtain asepsis and the primary union of the wound; for such experiments will often fail unless the antiseptic or aseptic precautions are successful. The technique of the operation he proposes is as follows:

When the patient is etherized the outline of the posterior border of the glans is marked on the skin with an aniline pencil. The mucous membrane is next cut away, leaving only a free edge of about one-eighth of an inch in width. Any bleeding that occurs should be entirely arrested, and asepsis should be insured by frequent sponging with carbolic or sublimate solutions. Numerous horse-hair stitches are then inserted, so as to bring accurately together the fresh-cut edges of the skin and mucous membrane, and subsequently, after a further sponging and drying, a piece of gauze two layers in thickness, and wide enough to reach from the root of the penis nearly to the meatus, is wrapped *loosely* around the penis, and secured by several applications of the collodion brush, so that the air is kept in motion, and the patient should not be allowed to recover from the anæsthetic until the dressing is quite firm and hard.

In this manner the penis is protected by a kind of carapace, and the patient is relieved from the pain and tenderness attendant upon contact with the bedclothes or other objects. In fact, the organ can be handled as if no operation had been performed. It is hardly necessary to add that erections, which are under the usual conditions so painful, can not occur.

The points to note are: 1. The operation must be aseptic. 2. The gauze should be applied *loosely*. The dressing can be made to extend, at the will of the operator, as far forward as the meatus; but half an inch from the corona is ample. If the dressing has been put on rather tight, and some swelling of the glans behind the dressing occur, the collodion dressing can be extended forwards as the urethea, or iced dilute subacetate of lead lotion may be used. For removing the dressing slit it up with a pair of blunt-pointed scissors. It is usually worn for about a week. As yet I have never found it necessary to apply it a second time.

